# Substitution of Formal and Informal Home Care Service Use and Nursing Home Service Use: Health Outcomes, Decision-Making Preferences, and Implications for a Public Health Policy

**DOI:** 10.3389/fpubh.2017.00297

**Published:** 2017-11-24

**Authors:** Chia-Ching Chen, Tetsuji Yamada, Taeko Nakashima, I-Ming Chiu

**Affiliations:** ^1^Department of Epidemiology and Community Health, School of Health Sciences and Practice, New York Medical College, Valhalla, NY, United States; ^2^Department of Economics and Center for Children and Childhood Studies, Rutgers University, The State University of New Jersey, Camden, NJ, United States; ^3^Department of Economics, Rutgers University, The State University of New Jersey, Camden, NJ, United States

**Keywords:** formal and informal home care, healthcare outcomes, health disparity, nursing home care

## Abstract

**Objectives:**

The purposes of this study are: (1) to empirically identify decision-making preferences of long-term health-care use, especially informal and formal home care (FHC) service use; (2) to evaluate outcomes vs. costs based on substitutability of informal and FHC service use; and (3) to investigate health outcome disparity based on substitutability.

**Methodology and data:**

The methods of ordinary least squares, a logit model, and a bivariate probit model are used by controlling for socioeconomic, demographic, and physical/mental health factors to investigate outcomes and costs based substitutability of informal and formal health-care use. The data come from the 2013 Japanese Study of Aging and Retirement (JSTAR), which is designed by Keizai-Sangyo Kenkyu-jo, Hitotsubashi University, and the University of Tokyo. The JSTAR is a globally comparable data survey of the elderly.

**Results:**

There exists a complement relationship between the informal home care (IHC) and community-based FHC services, and the elasticity’s ranges from 0.18 to 0.22. These are reasonable results, which show that unobservable factors are positively related to IHC and community-based FHC, but negatively related to nursing home (NH) services based on our bivariate probit model. Regarding health-care outcome efficiency issue, the IHC is the best one among three types of elderly care: IHC, community-based FHC, and NH services. Health improvement/outcome of elderly with the IHC is heavier concentrated on IHC services than the elderly care services by community-based FHC and NH care services.

**Conclusion:**

Policy makers need to address a diversity of health outcomes and efficiency of services based on providing services to elderly through resource allocation to the different types of long-term care. A provision of partial or full compensation for elderly care at home is recommendable and a viable option to improve their quality of lives.

## Introduction

The rapid increase in an aging population through a prolonged life expectancy combined with an increase in dependent elderly in Japan (1, 2) has caused a shortage of labor supply (a decrease in the labor force participation rate from 60% in 2010 to 54% in 2030), a deterioration of the pension system (from one elderly vs. 2.4 working age persons in 2012 to 1.2 persons in 2060), an upsurge in health-care costs (an increase in health spending in % of GDP from 9% in 2010 to 14% in 2020, and to 19% in 2030), and a change in elderly nursing care programs/policies (3). An imminent aging society compels many governments to change their policy orientation from institutional formal health-care settings to informal home health care because of the forthcoming rising of the elderly health-care costs, and long-term care financing (4–7). The Japanese government has recently emphasized and promoted a more community-based comprehensive care system with preventive care to mitigate the financing burden (8). For example, in 2000, the public long-term care insurance system was implemented to restrain the rapid increase in health-care expenditures, as well as to accommodate the rising demand for elderly care with an aging population (65+) from 23% in 2010 to 31% in 2025 (9). The major feature of this approach by the Japanese government is to reduce institutional long-term care and the pensions and to reduce the ever increasing long-term care financing (ever rising social security benefits including health and pensions from 21.8% in 2010 in GDP and 24.4% in 2025) by developing community-based comprehensive long-term care (i.e., formal home care and informal home care for the elderly in Table 1). This is imperative because of the tightening health-care economy in addition to a lack of human resources, long-term care facilities, and financial resources. Is a change in policy/program from institutional formal care to a community-based formal/informal care efficient and sufficient?

**Table 1:** Descriptive statistics of variables used in the present study.

Variables	Min.	Max.	Mean	SD
**Dependent variable**				
Community-based formal home care	0	24	0.020	0.598
It includes day-care services, short-stay services, and rehabilitation services at a health-care center with the following scale: minimum of 0 services to 24 services per month. Unit of value is number of times				
Informal home care	0	8	0.008	0.205
It includes physical care services, services of home chores, basing services, rehabilitation services, and nurse visits at home with the following scale: minimum of 0 services to 8 services per month. Unit of value is number of times				
Nursing home care use	0	1	0.012	0.110
1 = yes and otherwise = 0				
Formal and informal home care	0	1	0.018	0.132
1 = yes and otherwise = 0				
**Independent variables**				
Enabling factors				
Availability of care resources: availability of care resources by children because a work place provides care-leave days, 1 = yes, otherwise = 0	0	1	0.123	0.329
Private health insurance policy in addition to the national health insurance program: a person who has a private insurance policy in addition to the national health insurance program, 1 = yes and otherwise = 0	0	1	0.621	0.485
Accessibility of health-care services and facilities: it also includes accessibility to pharmacists, 1 = yes and otherwise = 0	0	1	0.854	0.352
Care-leave days by a worker for elderly parent(s)	0	60	0.214	2.214
Care-leave policy by a work place. 1 = yes and otherwise = 0	0	1	0.006	0.078
Reinforcing factors				
Marital status: married = 1 and otherwise = 0	0	1	0.776	0.416
Degree of own health care required level by government regulation: a degree of professional care requirement by government regulation with the following scale: 1 = independent, 2 = needs preventive care, 3 = least requirement of professional care, 9 = highest requirement of professional care because of physical and mental severity	1	9	1.104	0.797
Degree of spouse health care required level by government regulation: It is the same scale above	1	9	1.494	1.661
Predisposing factors				
Age (years)	52	80	67.45	6.841
Educational level as knowledge: education; 1 = elementary and middle school, 2 = high school, 3 = junior college, 4 = senior college, 5 = university, 6 = master, 7 = doctoral degree	1	7	2.877	1.431
Perception of family responsibility for elderly’s health care and nursing care: 1 = yes and otherwise = 0	0	1	0.407	0.491
Health risk and economic factors				
Change in health status: a change in health status compared to 1 year ago: 1 = excellent, 5 = worse	1	5	3.075	0.496
Preventive care: receiving preventive care services for the past year, 1 = yes and otherwise = 0	0	1	0.668	0.470
Days of hospitalization: number of hospitalization days for the past year, minimum = 1 day and maximum = 365 days	1	365	1.926	13.968
Mental aspects: i have been feeling depressed lately, I have been feeling lonely lately, etc., Each question has a 4-point scale (1 = none, 2 = 1–2 days, 3 = 3–4 days, and 4 = 5 days and more). Thirteen questions are added (minimum = 13 days and maximum = 50 days in total)	13	50	17.574	5.757
Income of a household head: annual income, a unit of value is 10,000 Japanese yen	0	1,800	583.031	61.337
Income (spouse): annual income, a unit of value is 10,000 Japanese yen	10	400	61.428	11.296
Savings: amount of saving in Japanese yen, a unit of value is Japanese 10,000 yen	0	3,000	33.90	205.552
Assets: amount of asset in Japanese yen, unit of value is Japanese 10,000 yen	0	3,000	61.37	264.094
Instrumental variables				
Preventive cost: annual expenses of preventive cares, a unit of value is 10,000 yen	0	18	0.36	1.219
Tooth: tooth treatment per year, a unit of value is number of visits	0	52	4.03	6.649
Caremother2_indep: good health status of spouse’s mother who is not a member of husband’s family	0	1	0.91	0.201

There is extensive literature documenting long-term care issues (3, 6, 10–16). However, there are few empirical works documenting elderly behavioral choices regarding the interaction among community-based formal/IHC and nursing home care (11–13, 15).

Van Houtven and Norton (11) demonstrated a simultaneous approach with instrumental variable estimation about elderly people and stated that IHC reduces formal health care of older adults. However, their findings do not clearly reveal the substitution between informal and formal home care (FHC) and nursing home care. Yamada et al. (12) shows that the one-way substitution of informal home care for nursing home care and the existence of a weak two-way substitution between nursing home care and community-based day-service and short-stay facility centers in Japan using the General Survey on Actual Living Conditions of the Elderly data of 1990. Hanaoka and Norton (13) focus on the potential source, i.e., children, of informal care by using the data of the Nippon University Japanese Longitudinal Study of Aging in 2001. Their findings are that the benefits of having unmarried children compared to the opportunity costs of having children makes a difference in the use of nursing care. The approach by Sole-Auro and Crimmins (15) discloses the different influences of sociodemographic factors on formal and informal care for elderly persons in England, Spain, and the USA. The results show that the use of formal care is higher in England and the USA compared to Spain. However, functional limitation receiving care is higher in Spain.

### Purpose of This Study

The aforementioned studies do not provide clear-cut evidence to generalize a two-way substitution among informal home care, community-based formal care, and nursing home care. To our knowledge, very few or no study has been conducted to investigate “outcomes” vs. “costs” based on substitutability of informal and formal health-care use. The primary contribution of this paper is to detect decision-making preferences of long-term health-care use, especially informal and formal health-care use empirically. A crucial challenge is quantifying and evaluating outcomes vs. costs based on substitutability of informal and formal health-care use. Our critical second contribution is to investigate and clarify health outcome disparities based on substitutability among informal care, community-based formal care, and nursing home care.

## Materials and Methods

### Analysis

Increasing evidence has shown that decision-making preferences of substitutability between informal and formal health-care use (10, 14). Balia and Brau (14) not only underline statistically significant results but also emphasizes the size of substitution between nursing home care and community-based formal/informal care. Thus, in this study, the effects are divided into two dimensions.

**Figure 1:**
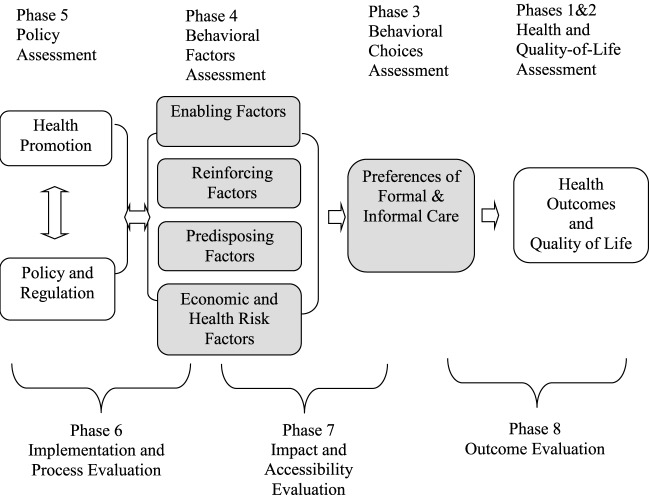
Application of PRECEDE–PROCEED model to examine preferences of formal and informal care and health outcome changes. Notes: (1) Green and Kreuter (18). (2) Glanz et al. (20). The Precede–Proceed model is a planning model used to analyze and assess a health behaviors and behavioral changes in the target population within a given socioeconomic and demographic environment. This model allows for a series of assessments and evaluations designed to help the health planning and policy and needs to the improvement of overall quality of life through the analysis of needs and problems.

The conceptual and theoretical framework underlying our research model is based on the PRECEDE–PROCEED model (hereafter referred to as the PP model) and postulates that health outcomes are attributed to the use of formal and informal care services based on behavioral, sociodemographic, and psycho-economics characteristics (17–19). The PRECEDE–PROCEED (PP) model is a well-known behavioral model. The model offers some concepts and analytical tools to help examine preferences of formal and informal long-term care for elderly people and health outcomes by using the Japanese Study of Aging and Retirement. By applying the PP model, we assess behavioral substitutability, which affects preferences for community-based informal care, formal care, and nursing home care by the elderly between Phases 3 and 4 in Figure 1. In addition, the study evaluates health outcomes from the preferences of formal and informal care services in health changes and quality of life of Phase 8. This study does not implement an intervention of health promotion, policy, and regulation in Phase 5.

There are four important categories of behavioral influential factors: enable, predisposing, reinforcing, and economics and health risk factors by controlling government regulations, sociodemographic, and psych-economic factor incorporating with the PP model framework. (1) Enabling factors include access to long-term care resources, availability of health care resources, health insurance, economic resources, social networks, development of skills, etc. This study also reflects financial burden, opportunity costs, and physical burden of informal care as a measure of enabling factors. (2) Reinforcing factors encompass the different kinds of rewards and feedback pertaining to formal and informal preferences. These factors can be derived from family, self, marital status, friends, and others who control the benefits and gains from community-based informal and formal care service use. These reinforcing behaviors comprise of different types of feedback and rewards after behavioral changes. (3) Predisposing factors of the PP model include personal attitudes, values, beliefs, knowledge, and perceptions. In this study, the PP model contains the following sociodemographic factors: gender, age 52 and older, education attainment, health knowledge, etc. Table 1 illustrates the characteristics of the aforementioned variables.

### Specification

The basic structural framework is shown in Figure 1. It is crucial to understand that decision-making preferences of long-term health-care use, especially informal and formal health-care use are attributed to the quality and quantity of physical family resources and community-based formal and informal programs, which can influence health outcomes and disparity as well as quality of life. The arrows of Figure 1 show that enabling, predisposing, reinforcing, economic, and health risk factors are predictors of preferences of IHC, community-based formal care, and nursing home care. As an exclusion criteria, this study focuses on elderly persons between the ages of 50 and 80. The following equations describe the basic structural model of analysis:

(1) $${\text{IHC}}_{\text{i}}=\alpha_{0}+{\text{E}}_{\text{i}}\alpha_{1}+{\text{R}}_{i}\alpha_{2}+{\text{P}}_{\text{i}}\alpha_{3}+{\text{X}}_{\text{i}}\alpha_{4}+\alpha_{5}{\text{LTC}}_{\text{i}}+\varepsilon_{\text{iIHC}}$$

(2) $${\text{FHC}}_{\text{i}}=\beta_{0}+{\text{E}}_{\text{i}}\beta_{1}+{\text{R}}_{\text{i}}\beta_{2}+{\text{P}}_{\text{i}}\beta_{3}+{\text{X}}_{\text{i}}\beta_{4}+\alpha_{5}{\text{LTC}}_{\text{i}}+\varepsilon_{\text{iFHC}}$$

(3) $${\text{and NH}}_{\text{i}}=\phi_{0}+{\text{E}}_{\text{i}}\phi_{1}+{\text{R}}_{\text{i}}\phi_{2}+{\text{P}}_{\text{i}}\phi_{3}+{\text{X}}_{\text{i}}\phi_{4}+\alpha_{5}{\text{LTC}}_{\text{i}}+\varepsilon_{\text{iNH}}$$

Equations 1–3 represent the relationship between the health behavioral choice of individual “i” and a person revealing the preference of health-care services, e.g., services of informal home care (IHC), services of FHC (FHC), and services of nursing home (NH). Factors will influence an individual’s preference of services and ε_i_IHC is an unobserved error, generally assumed to satisfy ƒ(ε_iIHC_|E, R, P, X) = 0. ε_i_FHC is an unobserved error, generally assumed to satisfy ƒ (ε_iFHC_|E, R, P, X) = 0. ε_i_NH is an unobserved error, generally assumed to satisfy ƒ (ε_iNH_|E, R, P, X) = 0. E_i_, R_i_, and P_i_ are vectors of the presumably exogenous variables of the predisposing, reinforcing, and enabling factors of individual i, respectively, that potentially influence health-care services. Equations 1–3 show the influential composition. Three different types of care, IHC, FHC, and NH, consisting of enabling (E), reinforcing (R), and predisposing (P) will influence preference of needed health care and are also incorporated in the extended PP model in Figure 1 to observe influential determinants. X_i_ is the vectors of additional determinants of service needs and economic and health risk factors. LTC represents FHC in Eq. 1, IHC in Eq. 2, and IHC or FHC in Eq. 3.

The association between informal home care services and community-based FHC services is an ambiguous association and could be positive or negative. To address this research question, i.e., the objectives, we hypothesize that home care and nursing home care is negative and both indicators are negatively and simultaneously determined within this study. An elderly person wants to have a certain preference under prevailing constraints, such as economic, psychological, social, and demographic factors, which are influenced by government policy and regulations, e.g., social security retirement benefits, i.e., income, welfare policy for elderly care, working regulation/condition for children who work and live with the elderly person, etc. Within the given constraints, a specific preference is determined by maximizing their own utility, i.e., satisfaction from services of home care or nursing home care.

The process and selection of receiving health-care services from home care or nursing home care will generate an economic, psychological, and physical burden on family members and spouses. This burden generally involves cost. For example, an increase in costs will distort the selection of needed health care and will be transformed into the pecuniary term of their own children and a spouse’s quality of daily life. This transformation or change depends on an elderly person’s psycho-economic and social factors. Constraints of prevailing human and financial resources will lead to an individual’s optimal decision.

### Data

This study uses the Japanese Study of Aging and Retirement (JSTAR), which was designed and carried out jointly by the Research Institute of Economy, Trade, and Industry and Hitotsubashi University in Japan, and the University of Tokyo. The JSTAR is a globally comparable data survey of the elderly, which is similar to the Health and Retirement Study (HRS) of USA. The design of JSTAR is like the U.S. HRS, the Survey of Health, Aging and Retirement in Europe (SHARE), and the English Longitudinal Study of Aging (ELSA). The 2011 wave was conducted in September and October and collected data on individual living circumstances of 4,500 persons aged between 50 and 80 years in three municipalities: Hiroshima city in Hiroshima prefecture, Chofu city in Tokyo, and Tondabayashi in Osaka.

The original sample size was 2,184 persons (983 men and 1,201 women; response rate of 48.5%). The survey has two units of observation: individual and household. A household is a single individual or individual with his/her spouse, whatever applicable. Unlike the HRS, the JSTAR only interviews one respondent per household but the survey includes several questions to the respondent about the spouse. The JSTAR covers a wide range of information including: income, wealth, working status, family structure, relationship with family members and neighborhood, capacity of memory and cognitive, health conditions, medical care, and nursing care.

### Statistical Analysis

Multiple-regression analyses are used to conduct and examine the effects of enabling, reinforcing, predisposing, and economic and health risk factors on home care and nursing home care. For this estimation, we used the methods of ordinary least squares (OLS) for home care regressions, a logit model for nursing home care, and the bivariate probit model to identify substitutability. The key dependent variables, which are shown in Table 2 measure the signs and the effects of the IHC: Eq. 1, the community-based formal care: Eq. 2, and the nursing home care by assuming some simultaneous effects in Eq. 3. We, therefore, implement to identify the exogeneity/endogeneity tests. This study uses the Hausman specification test to examine the endogeneity of this empirical model, and to examine preference of home care and nursing home care services.

**Table 2:** Empirical results of long-term care preference: informal home care (IHC) and community-based formal home care in Japan (OLS, *n* = 1,750).

Dependent variables	IHC		Community-based formal home care	
Variables	Coefficient	SE	Coefficient	SE
**Independent variable**				
Community-based formal home care IHC	0.071a	0.008	– 0.511a	– 0.063
**Independent variable**				
Enabling factors				
Availability of care resources	0.002	0.016	−0.008	0.043
Private health insurance policy in addition to the national health insurance program	0.004	0.010	0.017	0.028
Accessibility of health-care services and facilities	−0.171	0.014	−0.036	0.038
Reinforcing factors				
Marital status	0.006	0.012	0.008	0.034
Degree of own health care required level by government regulation	−0.001	0.006	0.001	0.017
Degree of spouse health care required level by government regulation	0.001	0.003	−0.006	0.008
Predisposing factors				
Age	0.002a	0.000	−0.001	0.002
Educational level as knowledge	0.006c	0.003	−0.000	0.010
Perception of family responsibility for elderly’s health care and nursing care	−0.018c	0.010	−0.039	0.028
Health risk and economic factors				
Change in health status	0.022b	0.010	0.023	0.027
Preventive care	−0.020c	0.011	−0.014	0.029
Days of hospitalization	0.001c	0.000	0.021a	0.001
Mental aspects	−0.001	0.001	−0.001	0.002
Income of a household head	0.000	0.000	−0.000	0.000
Savings	0.000	0.000	0.000	0.000
Assets	0.000	0.000	0.000	0.000
Number of observations	1,750		1,750	
*F*-statistics	5.93		21.62	
Probability of *F*-statistics	0.000		0.000	
*R*-squared	0.056		0.208	
Root MSE	0.211		0.567	

*Note: a, b, and c represent statistically significant level of regression coefficients as follows: 99% level (a), 95% level (b), and 90% level (c) for a two-tailed test*.

In order to address the outcomes and costs, the second objective, we used the OLS to examine opportunity costs of caring for an elderly person in terms of home care and nursing home care. As per the third objective of the study is to evaluate health outcome disparity, this study employed the concentration index (CI). The CI is used to quantify the degree of health outcome inequality in health outcomes. This analysis of IHC, FHC, and nursing home care is focused on horizontal equity and is not concerned with vertical issues (19, 21, 22). The aforementioned analyses involved phase 3, phase 4, phase 7, and phase 8 in Figure 1.

For this empirical study, there are three issues of reliability of estimation: exogeneity and endogeneity issue, multicollinearity issue, and heteroskedasticity in OLS and logit estimation. Health status of the elderly is included in a base specification and educational level, i.e., health knowledge. This is a factor that improves the efficiency with which the elderly can produce better health. The income level of the elderly affects the living standard, which contributes to their health. In addition, the correlation of educational attainment and income is generally positive. An elderly person with a higher education level is more likely to have higher health stock because of his/her health knowledge. Both variables are theoretically important to evaluate the elderly with preferences of health-care services with given resources, e.g., family human resources for elderly care. Therefore, we included multiple endogenous variables in our empirical analysis.

## Results

Table 2 represents the results from the OLS regression of the factors that are associated with preferences of the elderly for IHC and community-based FHC. All the results reported in Table 2 used heteroskedasticity-robust SEs, so, heteroskedasticity does not threaten the internal validity of the multiple-regression analysis with the definition of variables in Table 1. Table 3 offers the logit model results for examining a negative influence of informal and FHC services on nursing home cares. The results of Table 4 provide opportunity costs for elderly care through working hours by a family member.

**Table 3:** Empirical results of long-term care preference: nursing home (NH), informal home care (IHC), and community-based formal home care in Japan (logit, *n* = 1,750).

Dependent variables	NH		NH	
Variables	Coefficient	SE	Coefficient	SE
**Independent variable**				
Community-based formal home care	–	–	−1.247	224.39
IHC	−5.477	868.08	–	–
**Independent variable**				
Enabling factors				
Availability of care resources	0.122c	0.745	1.726b	0.732
Private health insurance policy in addition to the national health insurance program	−0.678	0.473	0.328	0.738
Accessibility of health-care services and facilities	−0.435	0.568	−0.455	0.757
Reinforcing factors				
Marital status	1.136	0.701	2.189c	1.128
Degree of own health care required level by government regulation	0.788a	0.095	1.048a	0.133
Degree of spouse health care required level by government regulation	−0.276	0.179	0.039	0.132
Predisposing factors				
Age	−0.124a	0.042	−0.020	0.055
Educational level as knowledge	−0.118	0.163	0.115	0.207
Perception of family responsibility for elderly’s health care and nursing care	0.165	0.453	−0.744	0.663
Health risk and economic factors				
Change in health status	−0.814c	0.465	−0.209	0.657
Preventive care	−0.507	0.472	1.241	0.832
Days of hospitalization	−0.185	0.367	−0.011	0.091
Mental aspects	0.004	0.038	−0.174c	0.007
Income of a household head	−0.002	0.006	−0.003	0.007
Savings	−0.001	0.002	−0.002	0.007
Assets	−0.001	0.001	−0.000	0.001
Number of observations	1,750		1,750	
Log likelihood	−101.342		−58.846	
Log likelihood chi square	108.83		127.27	
Prob > chi square	0.000		0.000	
*R*-squared	0.349		0.519	

*Notes: an SE of coefficient leads to *z* value (Wald statistic) by STATA. a, b, and c represent a confidence level of regression coefficients as follows: 99% level (a), 95% level (b), and 90% level (c) for a two-tailed test*.

**Table 4:** Empirical results of working hours and elderly care (ordinary least squares, *n* = 47).

Dependent variables	Working hours				
Variables	Coefficient	SE	*t*-Statistics	(95% confidence intervals)	
**Independent variable**					
Care time	22.907b	10.198	2.25	2.23	43.56
**Independent variable**					
Enabling factor					
Private health insurance policy in addition to the national health insurance program	2.325	6.732	0.35	−11.31	15.96
Reinforcing factor					
Marital status	11.983c	6.607	1.81	−1.40	25.37
Predisposing factors					
Age	0.559	0.566	0.39	−0.58	1.70
Educational level as knowledge	−4.085c	2.242	−1.82	−8.62	0.45
Health risk and economic factors					
Change in health status	−1.211	2.984	−0.41	−7.25	4.83
Income of a household head	−0.287b	0.116	−2.46	−0.52	−0.05
Income (spouse)	−0.036	0.051	−0.71	−0.13	0.06
Constant	147.95	93.341	1.59	−41.17	337.07
Number of observations	47				
*F*-statistics	2.41				
Probability of *F*-statistics	0.029				
*R*-squared	0.3691				
Root MSE	19.064				

*Note: a, b, and c represent statistically significant level of regression coefficients as follows: 99% level (a), 95% level (b), and 90% level (c) for a two-tailed test*.

Table 5 presents the results of a bivariate probit model to examine a substitutability of informal and formal health-care use and Institutional care.

**Table 5:** Empirical results of long-term care preference: formal and informal home care (IHC) and institutional care (bivariate probit, *n* = 1,694).

Dependent variable: the 2nd stage of bivariate probit model	Institutional care (nursing home care)			
Variables	Coefficient	SE	*z*	*P* > |*z*|
**Independent variable**				
Enabling factors				
Availability of care resources	−52.324	53,448.2	−0.000	0.999
Private health insurance policy in addition to the national health insurance program	0.137	0.328	0.420	0.676
Accessibility of health-care services and facilities	−0.296	0.343	−0.860	0.387
Care-leave days by a worker for elderly parent(s)	−4.214	12,151.0	−0.000	1.000
Care-leave policy by a work place	0.674	1.105	0.610	0.542
Reinforcing factors				
Marital status	0.768	0.681	1.130	0.259
Degree of own health care required level by government regulation	0.484	0.073	6.620	0.000
Degree of spouse health care required level by government regulation	0.013	0.067	0.200	0.843
Predisposing factors				
Age	−0.029	0.023	−1.230	0.217
Educational level as knowledge	0.103	0.095	1.080	0.280
Perception of family responsibility for elderly’s health care and nursing care	0.372	0.284	1.310	0.191
Health risk and economic factors				
Change in health status	−0.116	0.335	−0.350	0.729
Preventive care	0.607	0.409	1.480	0.138
Days of hospitalization	−0.004	0.040	−0.110	0.913
Mental aspects	−0.095	0.046	−2.060	0.039
Income of a household head	−0.001	0.003	−0.340	0.732
Income (spouse)	−0.005	0.002	−1.980	0.047
Assets	0.001	0.001	0.180	0.853
Constant	2.638	3.309	0.800	0.425

**Dependent variable: the first stage of bivariate probit model**	**Formal and IHC**			
**Variables**	**Coefficient**	**SE**	***z***	***P* > |*z*|**

**Independent variable**				
Enabling factors				
Availability of care resources	0.094	0.053	1.760	0.078
Private health insurance policy in addition to the national health insurance program	−0.329	0.204	−1.620	0.106
Accessibility of health-care services and facilities	−0.269	0.247	−1.090	0.277
Care-leave days by a worker for elderly parent(s)	0.061	0.035	1.720	0.085
Care-leave policy by a work place	−5.475	19,402.6	−0.000	1.000
Reinforcing factors				
Marital status	0.798	0.406	1.970	0.049
Degree of own health care required level by government regulation	0.423	0.054	7.780	0.000
Degree of spouse health care required level by government regulation	−0.135	0.088	−1.540	0.124
Predisposing factors				
Age	−0.052	0.017	−2.990	0.003
Educational level as knowledge	−0.049	0.075	−0.660	0.509
Perception of family responsibility for elderly’s health care and nursing care	−0.160	0.201	−0.800	0.425
Health risk and economic factors				
Change in health status	−0.350	0.217	−1.600	0.109
Preventive care	−0.087	0.208	−0.420	0.676
Days of hospitalization	−0.075	0.144	−0.520	0.601
Mental aspects	0.002	0.017	0.120	0.905
Income of a household head	−0.001	0.003	−0.240	0.814
Income of a spouse	−0.001	0.001	−0.140	0.890
Assets	0.001	0.001	−0.650	0.515
Constant	2.226	2.437	0.910	0.361
Number of observations	1,694			
Wald chi^2^ (36)	151.99			
Log likelihood	−139.64			
Prob > chi^2^	0.000	0.218		
Rho (ρ)	−0.734			
LR test of rho = 0: chi^2^(1)	6.927			
Prob > chi^2^	0.008			

*Notes: Rho (ρ) of IHC on nursing home care presents the estimate: −0.725 and SE: 0.220. The LR test is conducted to test the null hypothesis of rho = 0. The chi-squared test statistic with one degree of freedom is 6.613 and the corresponding *p*-value is 0.010, which rejects the null and indicates the significance of parameter rho*.

### Results of Reliability/Specification of Estimation

Regarding the issue of exogeneity/endogeneity, the study uses the Hausman specification/simultaneity test to examine the endogeneity of this empirical model: IHC, community-based FHC, and nursing home care are implemented. Under the null hypothesis, there is no simultaneity and correlation between, IHC variable, and ε_iNH_, which an error term of NH Eq. 3, and community-based FHC variable and ε_iFHC_, which is an error term of NH Eq. 3 should be 0, asymptotically. The predicted variable of IHC or FHC with instrumental variables is included in the structural form. The study used three instrumental variables: expenses of preventive care, tooth treatments, and health status of spouse side of parent in Table 1. For Hausman specification/simultaneity test, two of the predicted variables in the structural equations were not found to be statistically significant at the 5% level, the predicted variable of IHC (coefficient = −0.0449; SE= 0.1790; *t* = 0.25, and *p* > |*t*| = 0.802) in the NH regression, and the variable of community-based FHC (coefficient = −0.0384; SE = 0.4804; *t* = 0.08 and *p* > |*t*| = 0.936) in the NH regression. The results imply both IHC and community-based FHC variables are exogenous (the outcome available upon request).

The hypothesis that the coefficient on the health status, the coefficient on education, and the coefficient on the income are zero is an example of a joint hypothesis on the coefficients in the multiple regressions (1, 2, and 3). The regressors are possibly multicollinear and linear relationship among some or all-explanatory variables of a regression model makes precise estimation difficult. The variance inflation factors (vif) for the obese and overweight groups’ regressions range from 7.04 to 8.25 in Table 2. All vifs are less than 10. As a rule of thumb, when analyzing standardized data, a vif < 10 indicates a non-harmful multicollinearity (23).

### Results of Informal and FHC Use and NH

The results show that the influence of both coefficients of community-based FHC on IHC (second column) and the effect of informal care (fourth column) on community-based home care are statistically significant and positive in Table 2. One-unit (10 units) increase in community-based FHC services received by an elderly person will raise 0.071 (0.71 units) units of IHC services. On the other hand, one unit (10 units) increase in IHC services received by an elderly person will raise 0.511 (5.11 units) of community-based FHC services.

In Table 2, an elderly person who has health related knowledge will have preventive care. The coefficient of preventive-care service use (−0.020 in the second column) in *Health risk and economic factors* has less use of IHC series. The results state that elderly people are healthier by using preventive care than those who do not have preventive care. Other interesting results are that the effects of “Days of hospitalization” on both IHC (0.001) and community-based FHC (0.021) are positive and statistically significant. One-day hospitalization (10 days) will raise 0.001 (0.01) unit increase in IHC services. The services of community-based health-care center include day-care services, short-stay services, and rehabilitation services at a health-care center. The effect of hospitalization on the community-based home care center is that an increase in 1-day hospitalization by an elderly person will raise 0.021 units increase in services at the community-based FHC center.

For the results of NH with a logit method, both effects of community-based FHC and IHC are negative but not statistically significant in Table 3. Analytical model of 3 in *Specification* correspondingly proposes “additional examinations” in the NH about a matrix of Pearson correlation, using observation with no missing values and showing probabilities from “*t*-tests” of H_0_: *p* = 0 on each correlation. The results of Pearson correlations: IHC vs. NH (−0.0047, *t* = 0.8356) and HC vs. NH (−0.0038, *t* = 0.8669). Both are weakly and negatively correlated and both results are not statistically significant. For the variable “Degree of own health care required level by government regulation” holding the other influential factors constant, the log of the odds in favor of nursing care increases by 0.788%. A rise of one degree of professional care requirement by government criteria for an elderly with IHC raises 1.048% for an elderly with community-based FHC in Table 3.

Focusing on the relationship and substitution between IHC and community-based FHC, and NH, we implement “bivariate probit model” in Table 5. The new information provided by the bivariate probit model is the estimate of “ρ(rho),” the correlation coefficient for the two error terms. Table 5 shows that the dependent variable of the first stage use IHC and FHC (informal and FHC) and the second stage use NH nursing home care with the same independent variables for both regressions. The estimate of FHC and NH is “ρ” = −0.734 and Likelihood ratio test of “ρ” with chi-squared test of 6.927 with prob > chi-squared teat = 0.000; and the estimate of IHC and NH is “ρ” = −0.725 and Likelihood ratio test of “ρ” with chi-squared test of 6.613 with prob > chi-squared test = 0.010. Both estimations are significantly different from 0. The results indicate that unobservable factors are positively related to IHC and community-based FHC, but negatively related to NH. These empirical results do not provide quantitative clear-cut evidence about the substitution of IHC and community-based FHC for nursing home cares.

These are plausible results that indicate that unobservable factors are positively related to IHC and community-based FHC, but negatively related to NH. These empirical results do not provide a quantitative clear-cut evidence about the substitution between IHC and community-based FHC, and NH.

### Results of Opportunity Costs for Caring for the Elderly As IHC

We notice that there might be significant missing value because the age ranges from 50 years old (minimum) and 80 years old (maximum) in Table 1. When an elderly father or mother needs help, his or her children become caretakers. Thus, we imposed an exclusion criterion. Table 4 shows the sample size is *n* = 47. This number still generalizes the outcomes of these empirical results (23). This study provides new insight of IHC concerning opportunity cost for elderly care. The results of Table 4 evaluate costs of elderly care, which represent working hours by a family member. The coefficient of “care time” is a reciprocal specification to understand the effect and an optimal care-day off from work by a family member for an elderly care at home. The effect of care time by a family member in the second column in Table 4 is that a 1-day increase in elderly care days on overage per year will reduce 22.907 h of work hours on average.

Interesting results are other influential variables such as marital status, educational level, and income. A married person works 11.983 h per month more than an unmarried person. The result means that a spouse can take care for an elderly person and will raise husband economic activities. Both variables: education and income are theoretically important to evaluate the cost of activities by the elderly’s children who takes care of a father or mother at home. One level increase in education level will reduce working hours of the elderly’s child, who lives with an elderly parent, by 4.085 h (the second column in the predisposing factor).

## Discussion

### Elderly Care by Informal and FHC Use and NH, and with Opportunity Costs

The primary parameter of interest in this study is the influence of behavioral preferences or choices of home care or nursing home care. Van Houtven and Norton (11) validate that IHC reduces formal health care of old adults. Their findings do not clearly reveal the substitution of IHC and FHC services for nursing home care services. However, Yamada et al. (12) express one-way substitution of IHC for nursing home care, but a weak two-way substitution between nursing home care and community-based day-service. Hanaoka and Norton (13) emphasize children, especially unmarried children, affect children’s opportunity costs for choosing and using of nursing care. Sole-Auro and Crimmins (15) do not clearly focus on use of formal and informal care. We try to fill the gap of aforementioned findings.

Unlike previous studies, the IHC service users tend to use more community-based FHC services in Japan. Although the size of effects is different, both services complement each other. Similarly, the impacts of both services are that a 1% increase in community-based FHC will affect IHC services to rise by 0.18%. A 1% increase in IHC service will affect community-based FHC services to rise by 0.22%. The elderly person after hospitalization commonly receives health-care services at the community-based FHC center.

Yamada et al. (12) illustrate that the one-way substitution of IHC for nursing home care and the existence of a weak two-way substitution between nursing home care and community-based day-service and short-stay facility centers in Japan. Our study of logit result shows the effects of community-based FHC and IHC services on nursing home care services are negative but not statistically significant and this study is not able to reconfirm the empirical study by Yamada et al. (19). However, using “bivariate probit model,” we examined the relationship and substitution between IHC and community-based FHC, and NH, our results indicate that unobservable factors are positively related to IHC and community-based FHC, but negatively related to nursing home care. These empirical results some support that the substitution of IHC and community-based FHC services for nursing home care services.

The coefficient represents the maximum limitation of working hours is 147.95 h under the current family environment with controlling other sociodemographic and economic factors. By using the mean of annual income divided by working hours assuming 40 h per week, our estimation shows that wage per hour is 3,020.72 in Japanese yen. Using the coefficient of 22.907 h, we calculate a monthly opportunity cost is about 69,197 in Japanese yen which is about $692 ($1 = 100 Japanese yen as the current exchange rate for simplicity). The effect of income on the elderly care by a family member is that an increase in 10,000 in Japanese yen will lower 287 h of working hours by the elderly child with an elderly parent.

Health knowledge and behaviors are important elements of health and health behaviors. Health knowledge based on education profoundly influences the daily lives of the elderly. The earlier study of health knowledge disclosed how and whether an elderly person acquired health information and influenced the health behavior, health-care access, health outcomes, and quality of life (19). Our results do not reveal clear-cut evidences that the effectiveness of health education depends on the elderly’s beliefs regarding the importance of new information and on their confidence in their ability to change their own health behaviors. Moreover, improving the health knowledge and behaviors of the elderly is helpful in strengthening their ability for reasonable treatment, promoting the rational use of existing medical and health resources, enhancing their consciousness in disease prevention and self-health care, enabling the elderly to make correct judgments on their own health and dealing with public health emergencies scientifically.

### Disparity of Health Outcomes among IHC, FHC, and NH

**Figure 2:**
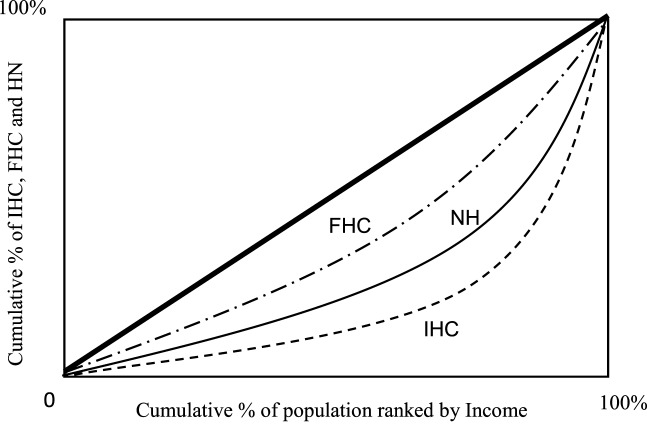
Concentration curves: informal home care (IHC), community-based formal home care (FHC), and Nursing home care (NH) by Income. Notes: concentration index (CI): CI using formula/covariance method. The CI of informal home care is 0.485c (0.10); the CI of FHC is 0.318c (0.08); and the CI of NH is 0.356b (0.04). SE: SEs of the CI using formula/covariance method. a, b, and c represent statistically significant levels of 99% level (a), 95% level (b), and 90% level (c) for a two-tailed test.

**Figure 3:**
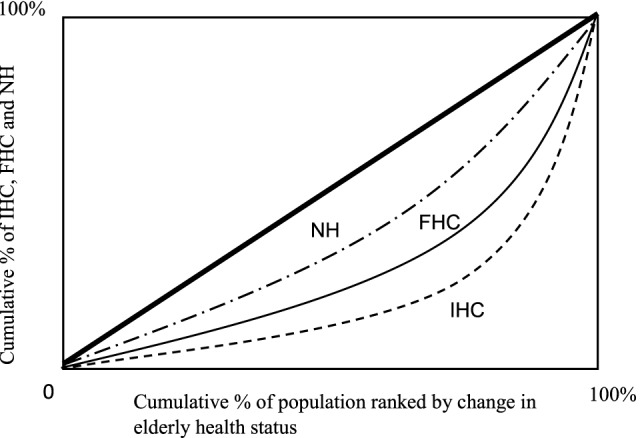
Concentration curves: informal home care (IHC), community-based formal home care (FHC), and Nursing home care (NH) by change in health status. Notes: concentration index (CI): CI using formula/covariance method. The CI of informal home care is 0.919b (0.04); the CI of FHC is 0.874c (0.08); and the CI of NH is 0.670c (0.06). SE: SEs of the CI using formula/covariance method. a, b, and c represent statistically significant levels of 99% level (a), 95% level (b), and 90% level (c) for a two-tailed test.

**Figure 4:**
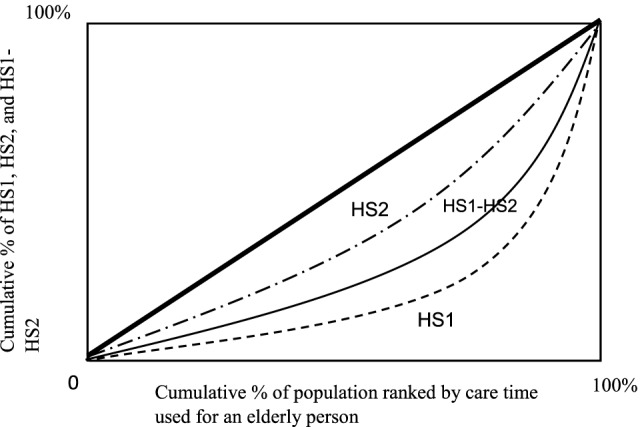
Concentration curves: original health status (HS1), current health statue (HS2), and its difference (HS1-HS2) by care time used for an elderly person. Notes: concentration index (CI): CI using formula/covariance method. The CI of informal home care is 0.919b (0.04); the CI of FHC is 0.874c (0.08); and the CI of NH is 0.670c (0.06). SE: SEs of the CI using formula/covariance method. a, b, and c represent statistically significant levels of 99% level (a), 95% level (b), and 90% level (c) for a two-tailed test.

Figures 2–4 show the concentration curves (CC), which emphasize and measure health inequalities and identify inequalities in health by using the CI. The index is negative when the curve is above the equality line and positive when the curve is under the equality curve. The CI is defined regarding the CC, which graphs on the *x*-axis the cumulative percentage of the population ranked by income beginning with the lowest, and on the *y*-axis the cumulative percentage of “IHC, FHC, and HN care” (19, 21, 22).

#### Income

In Figure 2, CI is defined as the CC, which is graphed on the horizontal axis as the cumulative percentage of the population ranked by income beginning with the lowest, and on the vertical axis as the cumulative percentage of IHC, community-based FHC, and NH by corresponding to each cumulative percentage of the population of the income. The CI is positive when the CC lies below the diagonal and negative when it lies above. The lowest value of the CI, −1, implies that all the elderly care is concentrated in the lowest level of household income. The maximum value of the CI, +1, implies that elderly care is concentrated in the highest level of household income. Figure 2 presents that the CC of the IHC, and the informal home care burden is concentrated more heavily and unevenly amongst IHC family/person relative to NH family/person as the NH as the second and community-based FHC family/person of the FHC. All CIs are statistically significant, and, interestingly, an elderly care with IHC is more concentrated in high income households, while an elderly care with FHC is more evenly concentrated in different income levels. The indices of Figure 2 show the value of the CIs measure for the disparity of elderly care by the IHC, the NH and the FHC.

#### Change in Health Status

For Figure 3, the magnitude of a relative disparity depends on the magnitude of the reference point from which the disparity was measured. The CC of IHC is farthest from the line of equality. The elderly care with health of elderly by NH is the nearest to the line of equality. These results demonstrate very interesting health issues about outcomes. The health variable is a change in health level/status of elderly from health level/status in period 1 to the health in period 2. A large change reveals better health improvement, i.e., more efficient elderly care. Regarding health outcome efficiency issue, the IHC is the best one among three types of elderly care: IHC, FHC, and NC since the IHC concentrates on largely at the left side of the health of the *x*-axis.

The size of an absolute disparity among the elderly care: IHC, FHC, and NH is expressed by the index in terms of each type of care of influence measured by the shape of the curve pm Figure 3. The CI of IHC is 0.919b (0.04); the CI of FHC is 0.874c (0.08); and the CI of NH is 0.670c (0.06). All CCs are positive and the value of the CI of IHC is larger than the FHC and the HN. Health improvement/outcome of elderly care by the IHC is heavier concentrated on IHC services than the elderly care services by community-based FHC and nursing home care services.

#### Care Time

Figure 4 presents that the CC of care time for the elderly at the original health status (HS1) is lowest and heavily concentrated on the left of care time among three measures (HS1, HS2, and HS1-HS2). The HS2 denotes the second period of health status. The difference is HS1-HS2 and the larger difference requires disproportionate concentration toward right. The HS1 is concentrated more heavily and unevenly among HS1 elderly relative to the elderly of HS2. It implies that after improving health status with care, the elderly of health status HS2 needs less care services and care time is more evenly distributed in different care time levels. All CIs are statistically significant, and, interestingly, the health gap between HS1 and HS2 is more evenly concentrated than HS1 in different care time levels. The larger difference of HS1-HS2 is less efficient outcome level of health than the small difference. The indices below Figure 4 show the value of the CIs measure for the disparity of health with care time among the categories of HS1, HS2, and HS1-HS2.

### Summary, Policy Implications, and Limitations

There is little doubt that the rapid increase in aging population through a prolonged life expectancy with an increase in dependent elderly causes to reduce institutional long-term care to reduce ever increasing long-term care financing (4–7). Recent studies provide some opening evidence about empirical work documenting elderly behavioral choice regarding the interaction among community-based formal/informal home care and nursing home care by Van Houtven and Norton (11), Yamada et al. (12), Hanaoka and Norton (13), and Sole-Auro and Crimmins (15). However, evidence of differential outcomes has been inconclusive. In this study, we apply the theory-oriented empirical study in the health economics fields and attempt to explore the full range of factors and how preference of decision-making for IHC, community-based FHC, and NH contribute to the relationship between them, health outcomes, and health disparity, which is caused by receiving services from IHC, community-based FHC, and NH. This study tries to fill in a critical gap within the literature.

The statistics of this study illustrate that a 10-unit increase in community-based FHC services received by an elderly person will raise 0.71 units of IHC services while a 10-units increase in IHC services received by an elderly person will raise 5.11 units of community-based FHC services. We evaluate this relationship with an elasticity term, the effects of the both services are that a 1% increase in community-based FHC will affect IHC services to rise by 0.18% while a 1% increase in IHC service will affect community-based FHC services to rise by 0.22%.

The findings of this study confirm that the IHC and community-based FHC services are complements. To analyze the substitution effects between IHC and community-based FHC, and NH, we implemented “bivariate probit model.” The probit analysis reveals the negative relationship, i.e., substitution between home care services and nursing home care services as follows. The result of statistics test of “*p*” with chi-squared test of 6.927 with prob > chi-squared test = 0.00. Both estimations are significantly different from 0. These are plausible results that indicate that unobservable factors are negatively related to NH and home care services. This study does not provide a quantitative clear-cut evidence about the substitution between IHC and community-based FHC, and NH.

This study incorporates family care hours and wages to evaluate the opportunity costs, which is the loss of salary income. The effect of care time is that a 1-day increase in elderly care day on overage per year will reduce 22.907 h of work hours on average with the maximum limitation of working hours is 147.95 h under the current family environment. The result of the regression outcomes reveal that a monthly opportunity cost is about 69,197 in Japanese yen which is about $692 ($1 = 100 Japanese yen as the current exchange rate for simplicity).

An important contribution to the literature is that we integrate the analysis with the finding that the CC exposes that the care burden is concentrated more heavily and unevenly among IHC family/person relative to NH and community-based FHC family/person. Regarding health outcome efficiency issue, the IHC is the best one among three types of elderly care: IHC, community-based FHC, and NH services. Health improvement/outcome of elderly care by the IHC is heavier concentrated on IHC services than the elderly care services by community-based formal home care and nursing home care services.

Policy makers need to address a diversity of health outcomes based on providing services to the elderly through resource allocation to different types of long-term care: IHC, community-based FHC, and nursing home care services. The recent policy recommendation about a transition for delivery of long-term care services form an institutional care services to informal and formal community home care services and developments of community-based home care under the national health-care insurance program is feasible. Using a behavioral model (the PRECEDE–PROCEED model), we comprehend efficiency of health outcomes through IHC services relative to community-based FHC, which complements the IHC in this study. However, many families and caregivers give up their work efforts to take care of elderly parents. Loss of work efforts are their opportunity costs. A provision of partial or full compensation is recommendable and a viable option to improve their quality of lives since their loss of earing causes a deterioration of daily life to maintain quality.

Some of the limitations of this empirical study are as follows: First, a small sample size of elderly child who helps and supports elderly parents due to a large missing value; second, the long-term care selection/choice is a joint decision. However, the data do not clearly reveal/present this aspect; and the data do not include many samples of institutional elderly parents. Finally, an appropriate and more scientific sampling technique would have further improved the quality of data. Despite these limitations, this study contributes to the existing literature to fill the literature gap concerning long-term care services in terms of efficiency and health outcomes with health disparity, and loss of economic opportunity costs to take care for the elderly parents. The results of this empirical study shed light on aforementioned findings and related policy implications. Future research should develop and examine research on different societies and countries.

## Ethics Statements

Japanese Study of Aging and Retirement (the JSTAR) conducted the survey with the collaboration of the Research Institute of Economy, Trade and Industry (RIETI), Hitotsubashi University, and the University of Tokyo and distributed by the RIETI in Tokyo, Japan. Institutional review board approval is not required for this study because the data are a secondary analysis of de-identified JSTAR data.

## Author Contributions

C-CC, TY, TN, and I-MC all contributed to the study design, research question definition, analysis and manuscript drafting, and approved the final version. They all fulfill ICMEJ criteria for authorship since they all contributed to any significant intellectual content.

## Conflict of Interest Statement

The research was conducted in the absence of any commercial or financial relationships that could be construed as a potential conflict of interest.
